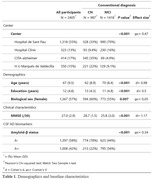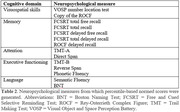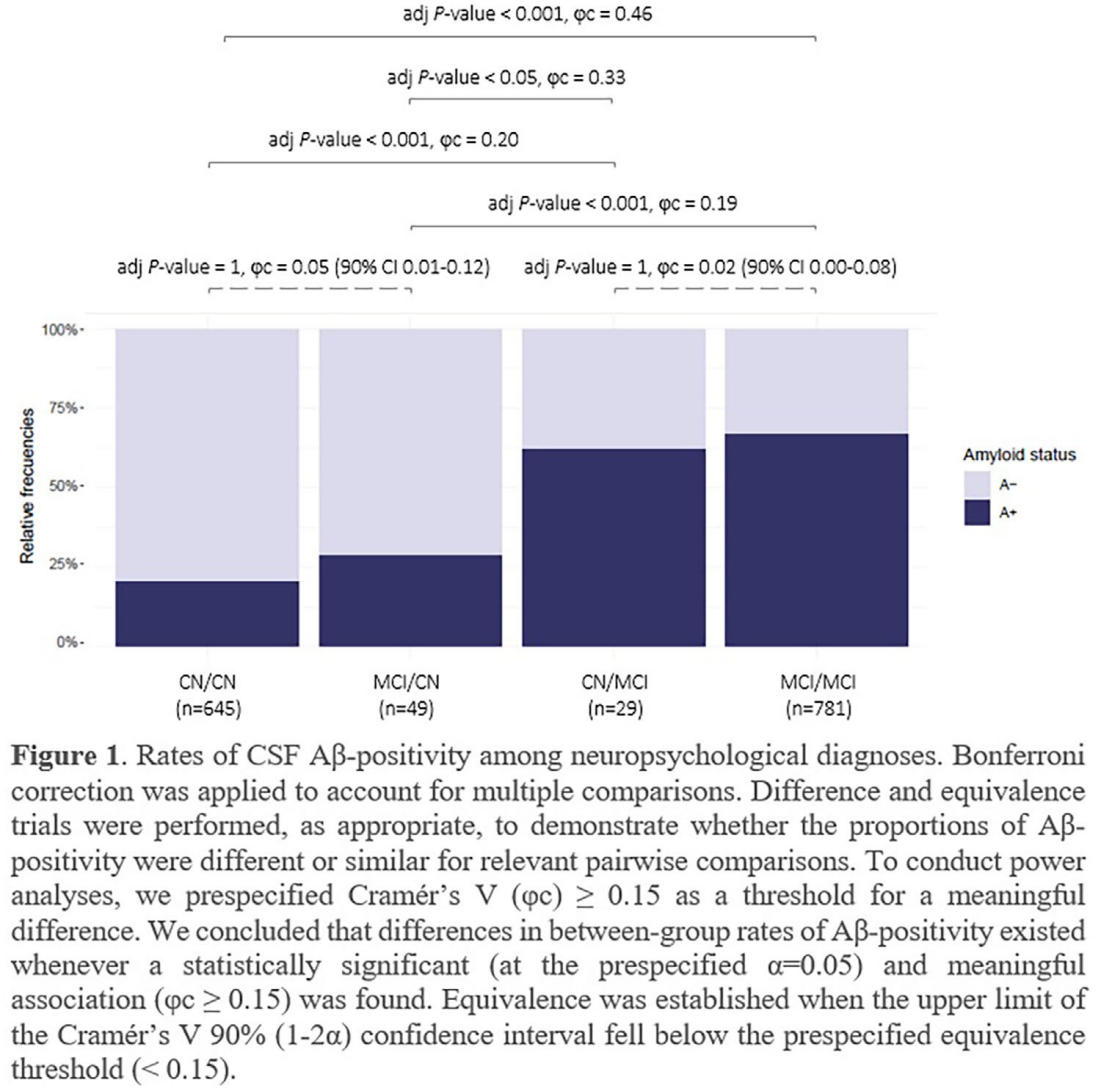# Added value of neuropsychological norms derived from amyloid‐negative cognitively normal adults

**DOI:** 10.1002/alz.091523

**Published:** 2025-01-03

**Authors:** Sara Rubio‐Guerra, María Belén Sánchez‐Saudinós, Isabel Sala, Alexandre Bejanin, Ainara Estanga, Mirian Ecay‐Torres, Carolina Lopez de Luis, Lorena Rami, Adrià Tort‐Merino, Magdalena Castellví, Ana Pozueta, María García‐Martínez, David Gómez‐Andrés, Carmen Lage, Sara López‐García, Pascual Sánchez‐Juan, Mircea Balasa, Albert Lladó, Miren Altuna, Mikel Tainta, Javier Arranz, Nuole Zhu, Daniel Alcolea, Alberto Lleo, Juan Fortea, Eloy Rodríguez Rodríguez, Raquel Sánchez‐Valle, Pablo Martínez‐Lage, Ignacio Illán‐Gala

**Affiliations:** ^1^ Hospital de la Santa Creu i Sant Pau ‐ Biomedical Research Institute Sant Pau ‐ Autonomous University of Barcelona, Barcelona, Catalonia Spain; ^2^ Fundación CITA‐Alzhéimer Fundazioa, Centro de Investigación y Terapias Avanzadas ‐ Osakidetza, Organización Sanitaria Integrada Debabarrena (OSI) ‐ University of Deusto, San Sebastián, Guipúzcoa Spain; ^3^ Hospital Clínic de Barcelona ‐ Fundació de Recerca Clínic Barcelona – IDIBAPS ‐ University of Barcelona, Barcelona, Catalonia Spain; ^4^ Hospital Universitario Marqués de Valdecilla – IDIVAL – University of Cantabria, Santander, Cantabria Spain; ^5^ Hospital Universitari Vall d’Hebron ‐ Vall d’Hebron Research Institute (VHIR) ‐ Autonomous University of Barcelona, Barcelona, Catalonia Spain; ^6^ Hospital de la Santa Creu i Sant Pau ‐ Biomedical Research Institute Sant Pau ‐ Autonomous University of Barcelona, Barcelona Spain

## Abstract

**Background:**

Neuropsychological performance guides diagnostic and therapeutic decision‐making on Alzheimer’s disease (AD) and related disorders. Despite broad recognition that amyloid‐beta (Aβ) impacts cognition during preclinical AD, the added value of Aβ‐negative norms remains uncertain. Furthermore, normative modeling is constrained by limitations inherent to traditional methods. Here, we derived next‐generation norms (NGN) for a comprehensive neuropsychological battery and compared their utility with that of traditional norms (TN).

**Methods:**

This multicenter study included 2405 non‐demented participants conventionally identified as cognitively normal (CN, n = 987) or with mild cognitive impairment (MCI, n = 1418) (**Table 1**). All participants had extensive neuropsychological data and CSF AD biomarkers at baseline. Based on the performance of 774 Aβ‐negative CN individuals, we derived NGN using generalized additive models for location, scale, and shape (GAMLSS). First, we compared the accuracy of NGN and TN to discriminate Aβ‐positive from Aβ‐negative MCI participants. Next, we actuarially reclassified all participants as CN or MCI according to both TN and NGN, and we examined the concordance of these classifications with AD biomarkers. For a subset of participants with clinical follow‐up (n = 1318), linear mixed‐effects modeling was used to capture the longitudinal change in Clinical Dementia Rating Scale‐Sum of Boxes. The Akaike Information Criteria (AIC) was employed to select the best model.

**Results:**

Age‐, education‐, and sex‐adjusted percentiles were obtained for 14 neuropsychological measures across main cognitive domains (memory, language, attention, executive functioning, and visuospatial skills) (**Table 2**). NGN outperformed TN at detecting prodromal AD for verbal memory measures, with all but one of the differences reaching statistical significance (p<0.05). Most participants were actuarially reclassified as either CN (43%) or MCI (52%) based on both TN and NGN. Among conflicting diagnoses (5%), the MCI/CN group (3%) ‐individuals diagnosed with MCI according to TN but considered CN as per NGN‐ was analogous to the CN/CN group, while the CN/MCI (2%) paralleled the MCI/MCI group in their rates of Aβ‐positivity (**Figure 1**). Cognitive status based on NGN better predicted clinical progression than TN (AIC = 5195.5 *vs* 5206.1, respectively).

**Conclusion:**

NGN incorporating Aβ status and GAMLSS yield stronger associations with biomarkers and disease progression than TN. This has direct implications for clinical practice and research.